# Identification of CTRP1 as a Prognostic Biomarker and Oncogene in Human Glioblastoma

**DOI:** 10.1155/2019/2582416

**Published:** 2019-04-30

**Authors:** Liyan Chen, Gang Su

**Affiliations:** ^1^Department of Nursing, The Fourth People's Hospital of Jinan, China; ^2^Department of Neurosurgery, The Fourth People's Hospital of Jinan, China

## Abstract

**Introduction:**

Glioblastoma (GBM) is the most frequent and malignant type of primary brain tumors in adults. The valuable prognostic biomarkers and therapeutic targets for GBM remain to be elucidated. The association of adipokines with cancer has been well documented. The C1q/TNF-related protein 1 (CTRP1), a novel adipokine, belongs to the CTRP family.

**Methods:**

In the present study, the expression and potential roles of CTRP1 in GBM were explored based on in silico evaluation, including GEPIA, the Pathology Atlas of the Human Protein Atlas, cBioPortal, TIMER, and SurvExpress. The CCK8, transwell, and wound healing assays were used to detect cell proliferation and migration.

**Results:**

It was found that mRNA expression levels of* CTRP1* were significantly upregulated in GBM tissues compared with those in nontumor tissues according to the analysis on public dataset and immunohistochemical results of GBM tissues (*P*<0.05). CTRP1 was mainly localized in the cytoplasm and cell membrane of GBM cells. The genetic alterations of CTRP1 occurred at a low rate in GBM (2 of 591 sequenced cases/patients, 0.33%). The mRNA expression levels of* CTRP1* were positively associated with the tumor-infiltrating macrophages and CCL2 in GBM (*P*<0.05, respectively). The higher mRNA expression levels of* CTRP1* were significantly correlated with higher risk and shorter overall survival time in GBM (*P*<0.05). CTRP1 knockdown significantly inhibited the proliferation and migration in human GBM cells, suggesting the inhibition of CTRP1 on human GMB progression. Moreover, CTRP1 knockdown inhibited CCL2 expression, and CCL2 overexpression reversed the inhibition of cell proliferation and migration induced by CTRP1 knockdown, suggesting that CTRP1 promoted tumor progression by regulating CCL2 expression.

**Conclusions:**

These findings suggest that CTRP1 potentially indicates poor prognosis in GBM and promotes the progression of human GBM.

## 1. Introduction

Glioblastoma (GBM) is the most frequent and malignant type of primary brain tumors in adults, which accounts for 15% of the latter. Despite of the extensive developments in surgery, radiotherapy, and chemotherapy during the past several decades, the median survival rate for patients with GBM is still very low [1, 2]. It is critical to identify new valuable prognostic biomarkers and therapeutic targets for GBM.

Adipokines, the multifunctional peptide hormones secreted by adipose tissue and other tissues, can play essential roles in energy balance, satiety, and immunity [3, 4]. The expression and functions of adipokines in the tumorigenesis and development have been a hot topic. Accumulating evidence has demonstrated that serum adipokines or tumor adipokines contribute to the proliferation, invasion/migration, angiogenesis, differentiation, and progression of various human cancers, including GBM [5–11]. In addition, they may act as valuable prognosis biomarkers in several tumors [12, 13].

The C1q/TNF-related protein 1 (CTRP1), a novel adipokine, is a member of the CTRP family and is widely expressed in various human tissues [14]. CTRP1 serves as a key regulator of glucose and lipid metabolism and is associated with atherosclerosis, coronary artery disease, and type 2 diabetes [14–17]. A total of 16 members of the CTRP family have currently been identified and they share a common structure [14]. Recently, CTRP3/CTRP4/CTRP6/CTRP8 were demonstrated to be engaged in tumorigenesis and progression by activating diverse signaling pathways [18–21]. Among them, CTRP8 can be expressed in human GBM cells and tissues and participate in regulating the motility and invasion ability of GBM cells [21]. CTRP1 has a close phylogenetic relationship and a high degree of sequence conservation with CTRP8 [22]. However, the expression and roles of CTRP1 in GBM remain unknown.

In the present study, the information of CTRP1 in GBM was explored using publicly available online datasets, including the expression level and location, the genomic alterations, the correlation with tumor-infiltrating macrophages and the potential underlying mechanism, and the prognostic value. The effect of CTRP1 on GBM cells was also investigated through the knockdown of CTRP1 in U87 and U251 cells.

## 2. Materials and Method

### 2.1. The mRNA Expression Analysis of CTRP1 in GBM and Nontumor Tissues

The mRNA expression level of* CTRP1* in GBM tissues (N=163) and nontumor tissues (N=207) was analyzed using the online database GEPIA (http://gepia.cancer-pku.cn/index.html). GEPIA is a novel interactive web server to analyze the RNA sequencing data based on TCGA and the GTEx projects [23]. The datasets (“TCGA tumors vs TCGA normal + GTEx normal”) were selected for the differential analysis. According to the instructions of GEPIA, one-way ANOVA was used for the differential analysis, and the disease state (Tumor or Normal) was used as a variable for calculating differential expression. A* P *< 0.05 was considered statistically significant.

### 2.2. The Protein Location Analysis of CTRP1 in GBM

The protein location of CTRP1 was analyzed using the Pathology Atlas of the Human Protein Atlas (HPA) (https://www.proteinatlas.org/pathology). The Pathology Atlas of HPA is an interactive open-access database containing the mRNA and protein expression data based on the integration of publicly available data from TCGA and data generated within the framework of the HPA. The expression and location of the proteins in patients with the respective cancer types including GBM are detected using a tissue microarray-based immunohistochemistry (IHC) analysis [24, 25].

### 2.3. The Analysis for Mutations and Putative Copy-Number Alterations from GISTIC

Genetic alteration frequency of* CTRP1* in GBM was analyzed in the TCGA dataset (Glioblastoma, provisional) (N=604) using cBioPortal (http://www.cbioportal.org/index.do) [26, 27]. The search was performed according to the online protocols of cBioPortal.

### 2.4. The Correlation Analysis

Tumor Immune Estimation Resource (TIMER) (https://cistrome.shinyapps.io/timer/) is an interactive web application for conveniently exploring and visualizing tumor immunologic and genomics data [28, 29]. The correlation of CTRP1 expression with the tumor-infiltrating macrophages levels in GBM was evaluated by the “gene” module of TIMER. The correlation of the mRNA expression levels between CTRP1 and CCL2 in GBM was explored by the “correlation” module of TIMER. A* P *< 0.05 was considered statistically significant.

### 2.5. Survival Analysis

The prognostic value of CTRP1 in the outcome of GBM was evaluated through the online system SurvExpress (bioinformatica.mty.itesm.mx/SurvExpress) [30]. The selected GBM database was Glioblastoma (TCGA) (N=538). The low-risk and high-risk groups were generated by the default prognostic index (PI). The Kaplan-Meier method and the log-rank test were used to estimate survival between the two risk groups. The Cox proportional-hazards regression for survival data was used to estimate hazard ratios. The box plot of CTRP1 expression by risk groups and the* P* value obtained from a* t*-test were also provided by SurvExpress. A* P *< 0.05 was considered statistically significant.

### 2.6. Immunohistochemistry

The tissue chip used in this study was purchased from Shanghai Outdo Biotech Co., Ltd. (China). Human tissues were stained using the EliVision™ plus kit (Maixin, China) according to the manufacturer's instructions. The CTRP1 immunostaining score was the sum of the staining intensity score and the positive staining cell rate score. The staining intensity was scored as follows: no staining: 0, weak staining: 1, moderate staining: 2, and strong staining: 3. The positive staining cell rate was scored as follows: 0 to 5%: 0, 5% to 25%: 1, 26% to 50%: 2, 51% to 75%: 3, and >75%: 4. A score below 2 points was considered to be CTRP1 negative expression and >3 points as CTRP1 high expression.

### 2.7. Cell Lines

Human glioblastoma cell lines, U87 and U251, were obtained from the Type Culture Collection of the Chinese Academy of Sciences. Cells were cultured in DMEM medium supplemented with 10% fetal bovine serum (FBS) and incubated at 37°C with 5% CO_2_. Cells were transfected with CTRP1 specific siRNA (siCTRP1) (Ruibo, China), negative control siRNA (NC) (Ruibo, China), or CCL2 overexpression plasmid (Ruibo, China) by Lipofectamine 2000 when having reached approximately 70% confluency.

### 2.8. Quantitative Real-Time PCR (qPCR)

Total RNA was resolved from U87 and U251 cells after transfection for 24 h using the TRIzol reagent and then reverse-transcribed to cDNA by the PrimeScript RT-PCR Kit (Takara Bio, Japan). The expression of* CTRP1* was performed by SYBR Premix Ex Taq II (TaKaRa, China) according to the manufacturer's instructions. The relative quantification of* CTRP1* was identified by the 2^−∆∆Ct^ method after being normalized to*β-actin*. Primers used were as follows:* CTRP1* sense: 5′-AAGGGCTCTGCTGGTCTTTC-3′, antisense: 5′-CCAGGTAGCCACTGAAGGTG-3′;* β-actin* sense: 5′-CCCGAGCCGTGTTTCCT-3′, antisense: 5′-GTCCCAGTTGGTGACGATGC-3′.

### 2.9. Western Blot

Total proteins from U87 and U251 cells were extracted by RIPA buffer after transfection for 48 h. Protein concentration was determined by the BCA protein assay kit (ComWin Biotech, Beijing, China). Cell lysates were separated by sodium dodecyl sulfate-polyacrylamide gel (SDS-PAGE) and transferred to a polyvinylidene difluoride (PVDF) membrane. Then, the membrane was blocked in tris-buffered saline (TBS) containing 5% nonfat milk for 2 h at room temperature and incubated overnight at 4°C with primary antibodies (anti-CTRP1, 1:1000; anti-GAPDH, 1:5000) (Proteintech Group, USA), followed by the secondary antibodies at room temperature for 1 h. The chemiluminescent signals of proteins were generated using the ECL reagent (Proteintech, USA) and quantified with Quantity One software.

### 2.10. CCK8 Assay

The proliferation of U87 and U251 cells was detected by Cell Counting Kit-8 (CCK-8). Cells were transfected with siRNA and then transferred into 96-well plates at 4000 cells per well. CCK-8 solutions were added to wells and cells were cultured for 2 h. Then OD values were measured on a microplate reader. Each experiment was performed in triplicate.

### 2.11. Transwell Assay

The transwell assay was performed to evaluate cell migration ability using a transwell chamber (Millipore, USA). Cells transfected with siRNA were seeded into a chamber. After being incubated for 48 h, cells migrated to the lower chamber were washed by PBS and stained with crystal violet for 10 min. Migrated cells were calculated from triplicate determinations.

### 2.12. Wound Healing Assay

5 × 10^5^ U87 or U251 cells were seeded in six-well plates for the wound healing assay. After transfection for 24 h, wounds were generated by a 10 *μ*l pipette tip. The wound was imaged after scratch for 24 h. ImageJ software was used for data analysis.

### 2.13. Statistical Analyses

In the present research, all experiments were repeated three times. SPSS 18.0 software was used for the statistical analysis. All data were expressed as means ± SD. One-way ANOVA analysis was used to assess differences between groups. Differences were considered statistically significant for values of* P*<0.05.

## 3. Results

### 3.1. The Expression Levels and Location of CTRP1 in GBM

**Figure 1:**
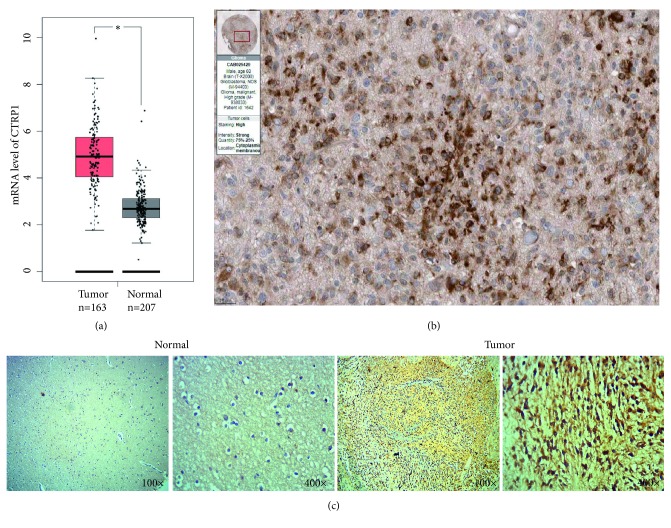
Overexpression of CTRP1 mRNAs and its protein location in GBM patients. (a) The box plot image showed mRNA levels of CTRP1 in GBM tissues (T) (N=163) and nontumor tissues (N) (N=207) from GEPIA (http://gepia.cancer-pku.cn/index.html). The datasets (“TCGA tumors vs TCGA normal + GTEx normal”) were selected for the differential analysis. According to the instructions of GEPIA, one-way ANOVA was used for the differential analysis, and the disease state (Tumor or Normal) was used as a variable for calculating differential expression. A* P *< 0.05 was considered statistically significant. (b) The immunohistochemical image of CTRP1 in Glioblastoma tissue from the Pathology Atlas of the Human Protein Atlas (https://www.proteinatlas.org/pathology). The patient ID was 1642. The scale bars were 25 um. (c) The expression of CTRP1 in glioblastoma and brain normal tissues was detected by immunohistochemistry. Photomicrographs were taken at 100x and 400x magnification, respectively. *∗P* < 0.05.

We first analyzed the expression of* CTRP1* on GEPIA. The log_2_ (TPM+1) was used for log-scale and |log_2_FC| cutoff=1. Based on the data from GEPIA, the mRNA expression levels of* CTRP1* in GBM tissues (N=163) were significantly higher than those in nontumor tissues (N=207) (*P*<0.05, Figure 1(a)). The IHC image of CTRP1 was obtained from HPA. The GB patient (ID: 1642) was male and age was 60. The image showed that the CTRP1 was localized in the cytoplasm and cell membrane of GBM cells. In GBM cells, the staining of CTRP1 was high, the intensity was strong, and the quantity ranged from 75% to 25% (Figure 1(b)). We further detected the differentiated expression of CTRP1 in 3 brain normal tissues and 30 human glioblastoma cases. As shown in Figure 1(c), the expression of CTRP1 in glioblastoma tissues was upregulated in comparison to normal tissues. Low expression was detected in all 3 cases of normal tissues, while 21 cases were highly expressed and 9 cases were lowly expressed in 30 cases of tumor tissues.

### 3.2. Genomic Alterations of CTRP1 in GBM

**Figure 2:**
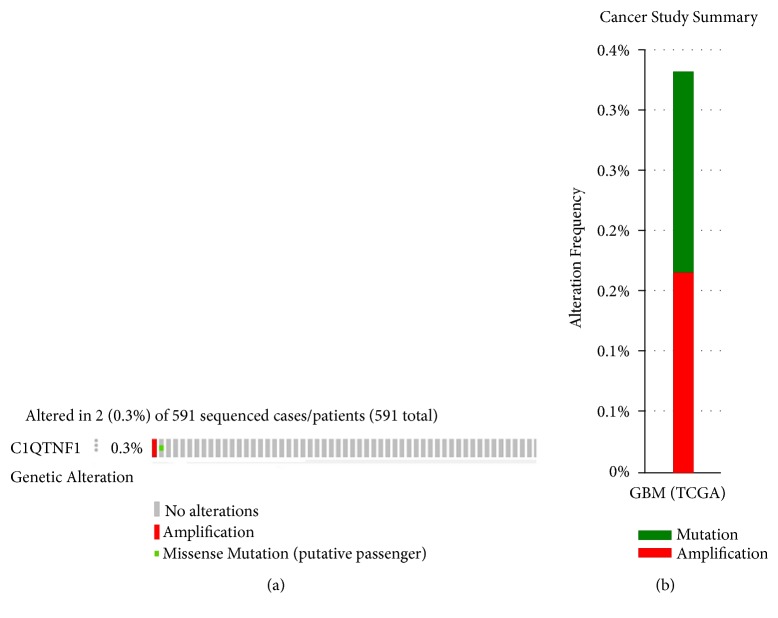
The genetic alteration of CTRP1 in GBM. (a) The proportion and distribution of samples with alterations in CTRP1 in GBM. The data were accessed using the online platform cBioPortal (http://www.cbioportal.org/index.do). The image was truncated on the right to exclude samples without alterations. (b) The cancer study summary of (a).

The data analyzed in cBioPortal showed that* CTRP1* altered in 2 (0.33%) of 591 sequenced cases/patients in the TCGA dataset (Glioblastoma, provisional, N=604) (Figure 2), which implied that the mutations or DNA copy-number alterations of CTRP1 occurred at a low rate in GBM.

### 3.3. The Correlation of CTRP1 Expression with the Tumor-Infiltrating Macrophages and CCL2 in GBM

**Figure 3:**
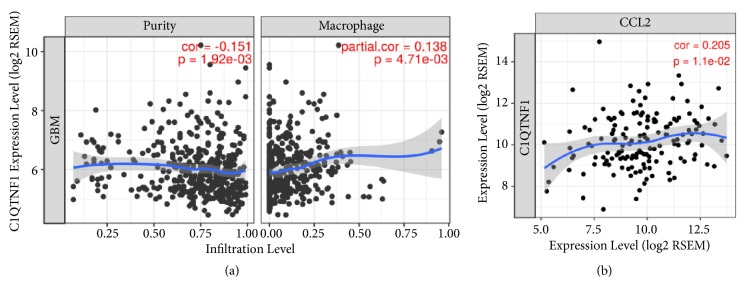
The correlation of CTRP1 expression with the tumor-infiltrating macrophages and CCL2 in GBM. (a) The correlation of CTRP1 mRNA expression with the tumor-infiltrating macrophages in GBM was analyzed using the “gene” module of TIMER (https://cistrome.shinyapps.io/timer/). (b) The correlation of CTRP1 mRNA with CCL2 mRNA in GBM was explored using the “correlation” module of TIMER. A* P *< 0.05 was considered statistically significant.

The data from the “gene” module of TIMER showed that* CTRP1* mRNA expression was correlated with the infiltration level of macrophages in GBM (partial. cor=0.138,* P*=4.71e-03) (Figure 3(a)). The data from the “correlation” module of TIMER identified the positive correlation of the mRNA expression levels between* CTRP1* and* CCL2* in GBM (cor=0.205,* P*=1.1e-02) (Figure 3(b)). When the tumor purity or age option was adjusted, the expression scatterplots between* CTRP1* and* CCL2* still had statistical significance (purity: partial. cor=0.19,* P*=2.60e-02; age: partial. cor=0.20,* P*=1.39e-02) (Figures S1 and S2).

### 3.4. The Prognostic Value of CTRP1 in the Outcome of GBM

**Figure 4:**
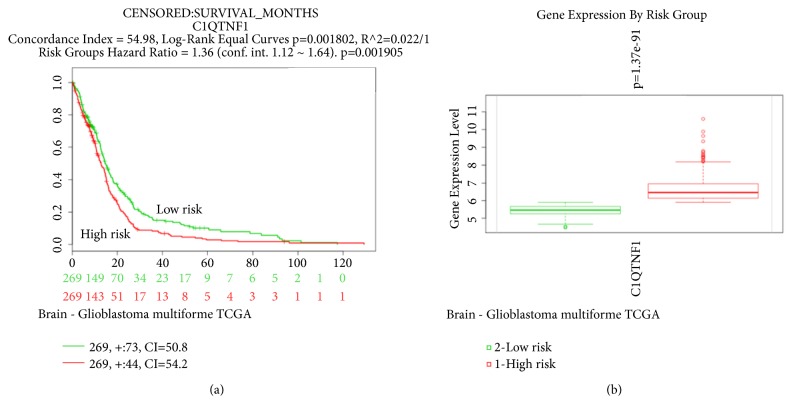
Evaluation of the prognostic value of CTRP1 mRNA expression in the TCGA dataset (Glioblastoma) in SurvExpress. (a) The patients from the TCGA dataset (Glioblastoma multiforme, N=538) in SurvExpress were divided into low- and high-risk groups according to the default prognostic index (PI). Survival differences between the two groups were demonstrated with Kaplan-Meier survival curves and censored data of overall survival were indicated. Green and red lines indicated low- and high-risk groups, respectively. (b) The mRNA expression level of CTRP1 in low- and high-risk groups. A* P* < 0.05 was considered statistically significant.

In SurvExpress, the patients from the TCGA dataset (Glioblastoma, N=538) were divided into low- and high-risk groups according to the PI. Survival difference between the two groups was demonstrated with Kaplan-Meier survival curves and censored data of overall survival (OS) were indicated. It was found that the OS of patients with high risk (red line) was significantly shorter compared with those with low risk (green line) (*P*<0.05) (Figure 4(a)). SurvExpress also provided the mRNA expression level of* CTRP1* in the two groups.  Figure 4(b) showed that mRNA expression of* CTRP1* in high-risk groups was significantly higher than that in the low-risk group (*P* <0.05). The data from SurvExpress implied that the higher expression of* CTRP1* was significantly correlated with higher risk and shorter OS time in GBM.

### 3.5. CTRP1 Knockdown Inhibited Proliferation and Migration in Human Glioblastoma Cells

**Figure 5:**
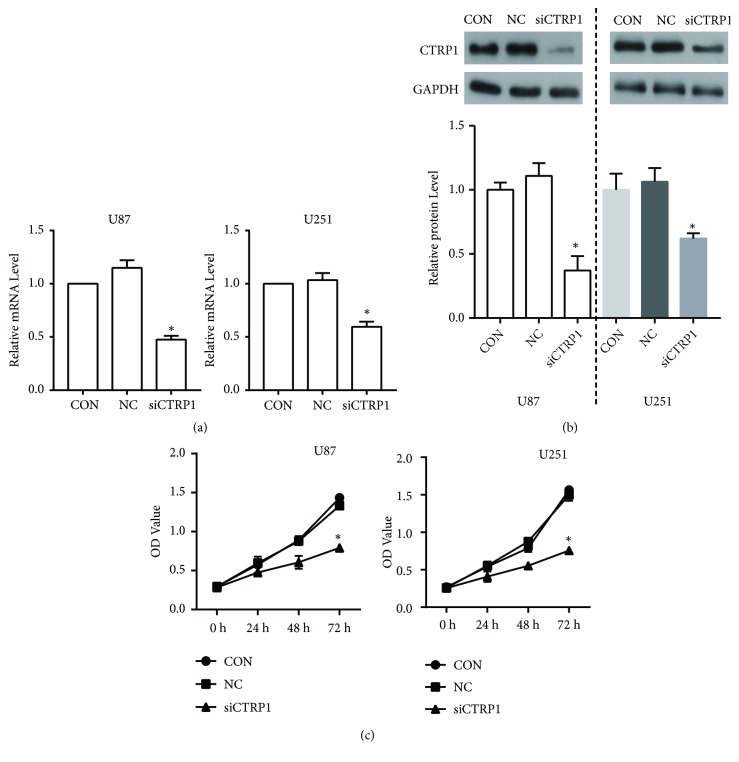
CTRP1 was overexpressed in human glioblastoma and siCTRP1 inhibited cell proliferation. (a) The expressions of CTRP1 in siCTRP1, NC, and CON cells were detected by qPCR. (b) The expressions of CTRP1 in siCTRP1, NC, and CON cells were detected by western blot. (c) The proliferation of siCTRP1, NC, and CON cells was confirmed by CCK8 assay. *∗P* < 0.05 vs. NC cells.

**Figure 6:**
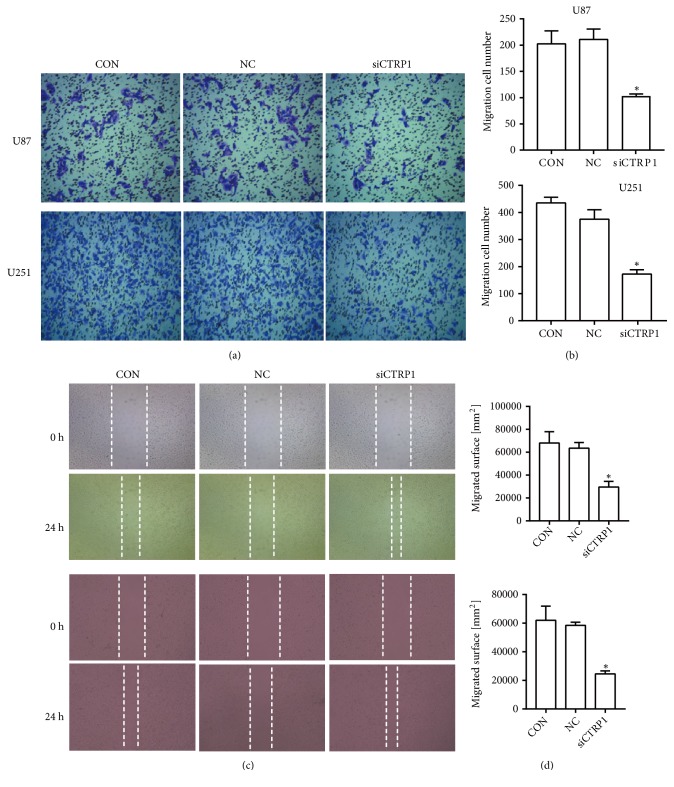
siCTRP1 inhibited the migration of U87 and U251 cells. (a) The migration of siCTRP1, NC, and CON cells was detected by the transwell assay. (b) The analysis of migrated cell number in the siCTRP1, NC, and CON groups. (c) The migration of siCTRP1, NC, and CON cells was detected by the wound healing assay. (d) The analysis of migrated cell number in the siCTRP1, NC, and CON groups. *∗P* < 0.05 vs. NC cells.

CTRP1 expression was inhibited by its specific siRNA (siCTRP1) to build the knockdown cells for elucidating its biological function in U87 and U251 cells. CTRP1 expression was significantly reduced in siCTRP1 cells compared with negative control (NC) and wild-type U87 control cells (CON) (Figures 5(a) and 5(b)). The CCK8 assay was performed to detect cell proliferation. After being transfected for 72 h, the OD value of siCTRP1 cells declined significantly, suggesting that siCTRP1 could inhibit the proliferation in U87 and U251 cells (Figure 5(c)). To further demonstrate the function of CTRP1 in human glioblastoma cells, we detected the migration of U87 and U251 cells in these three groups using the transwell and wound healing assays. As shown in Figures 6(a) and 6(b), the migrated cell number in the siCTRP1 group was declined significantly in comparison to the NC and CON group in both U87 and U251 cells. Similar results were also observed in the wound healing assay (Figures 6(c) and 6(d)). According to the above results, we predict that CTRP1 promotes tumor progression of human glioblastoma.

### 3.6. CTRP1 Knockdown Inhibited the Expression of CCL2 in Human Glioblastoma Cells

**Figure 7:**
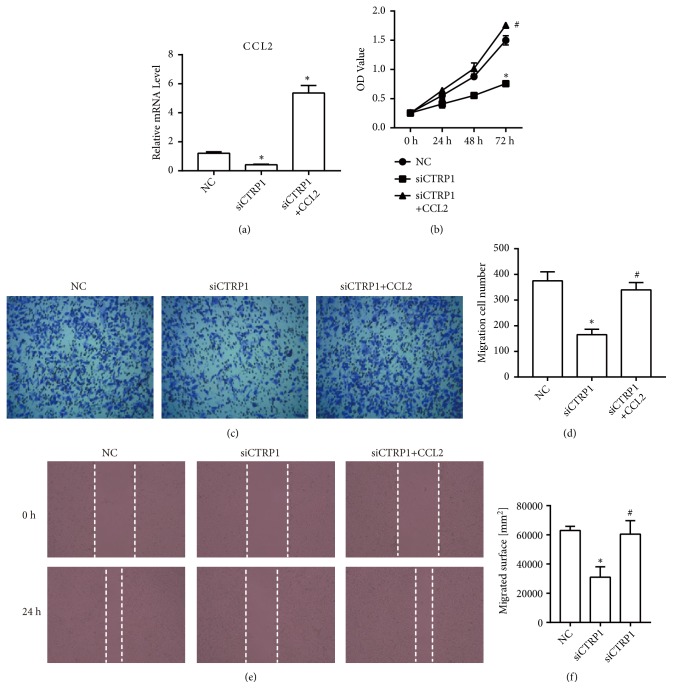
siCTRP1 inhibited the expression of CCL2 and the inhibition of proliferation and migration by siCTRP1 was reversed by the overexpression of CCL2. (a) The mRNA level of CCL2 was detected by qPCR. (b) The proliferation of U251 cells was confirmed by the CCK8 assay. (c) The migration of U251 cells was detected by the transwell assay. (d) The analysis of migrated cell number in U251 cells. (e) The migration of U251 cells was detected by the wound healing assay. (f) The analysis of migrated cell number in U251 cells. *∗P* < 0.05 vs. NC cells; #*P* < 0.05 vs. siCTRP1 cells.

We have found that there is a significant correlation between the expression of CTRP1 and CCL2 in human glioblastoma; thus we further investigated if knockdown of CTRP1 could influence CCL2 expression. The qPCR was performed to detect the mRNA expression level of* CCL2*. As shown in Figure 7(a), CCL2 expression was significantly suppressed by CTRP1 specific siRNA in U251 cells.

### 3.7. CCL2 Reversed the Inhibition of the Proliferation and Migration Induced by siCTRP1 in Human Glioblastoma Cells

To explore the effect of CCL2 on CTRP1 function in U251 cells, we overexpressed CCL2 in CTRP1 knockdown cells (siCTRP1+CCL2). The qPCR was performed to detect* CCL2* level in U251 cells (Figure 6(a)). The mRNA level of* CCL2* in cells transfected with CCL2 overexpression plasmid significantly increased compared with that in NC or siCTRP1 cells. Then, the proliferation and migration of each group were analyzed by the CCK8, transwell, and wound healing assays, respectively. From Figure 7(b), the OD value of CTRP1 knockdown cells with transfection of CCL2 overexpression plasmid markedly increased in comparison to CTRP1 knockdown cells. In addition, the transwell results showed a sharp rise in the migration cell number of siCTRP1+ CCL2 cells compared with that of siCTRP1 cells (Figures 7(c) and 7(d)). Similar results were also observed in the wound healing assay (Figures 7(e) and 7(f)). These results proved that CCL2 reversed the inhibition of the proliferation and migration induced by CTRP1 knockdown in U251 cells.

## 4. Discussion

The correlation of adipokines with cancer has been well documented [5–13]. The expression and the prognostic value of the novel adipokine CTRP1 in GBM were evaluated for the first time in the present study. It was found that CTRP1 located in the cytoplasm and cell membrane of GBM cells and its expression levels in GBM tissues were significantly higher than those in nontumor tissues. CTRP1 did not tend to be altered in GBM genome. CTRP1 expression was associated with the tumor-infiltrating macrophages and CCL2 in GBM. The higher expression of CTRP1 was significantly correlated with higher risk and shorter OS time in GBM. Our data in GBM provide novel insight linking CTRP1 with cancer.

Members of the CTRP family share the common structures, including a N-terminal signal peptide, a short variable region, a collagen-like domain, and a C-terminal globular C1q domain [14, 31]. Among them, CTRP8 was identified in primary GB cells, GB cell lines, normal astrocytes, and GB tissues using RT-PCR or IHC methods. CTRP8 can activate the leucine-rich G-protein-coupled relaxin receptor- (RXFP1-) dependent signaling pathways via the N-terminal “YAAFSVG” peptide motif, which can promote the motility and invasion ability of GB [21, 22]. However, there is no significant difference in CTRP8 mRNA expression between GBM tissues (N=163) and nontumor tissues (N=207) using GEPIA analysis (*P*>0.05, Figure S3). CTRP1 contains a close evolutionary relationship with CTRP8 and shares the N-terminal “YAAFSVG” peptide motif with CTRP8 or the peptides P59/P74 derived from CTRP8 [22]. Moreover, CTRP1 was also known as GIP (G-protein-coupled receptor-interacting protein) [32]. So we firstly analyzed the expression and location of CTRP1 in GBM tissues. It is widely accepted CTRP1 is a circulating protein [14, 33]. Meanwhile, previous study revealed that CTRP1 localized partly on the cell plasma membrane [32]. Consistent with the above study, CTRP1 was also found to be localized in the cytoplasm and cell membrane of GBM cells (Figure 1(b)). The mRNA expression levels of* CTRP1* in GBM tissues were significantly higher than those in nontumor tissues (*P*<0.05, Figure 1(a)). CTRP1 did not tend to be altered in GBM genome (Figure 2). Currently, information on the roles of CTRP1 in cancer and the receptor for CTRP1 remains limited. Whether the increase of CTPR1 in GBM tissues may activate RXFP1 receptors to promote tumorigenesis and the correlation of CTRP1 with clinicopathological parameters of GBM require in-depth studies in the future.

Recent studies showed that recombinant TNF-*α* and IL-1*β* enhanced the expression and secretion of CTRP1 in THP-1 cells and HUVECs [17]. CTRP1 expression was significantly increased during the differentiation of primary human macrophages from peripheral blood mononuclear cells [16]. CTRP1 treatment can facilitate the production of the chemokine (C-C motif) ligand 2 (CCL2, also known as MCP-1) belonging to the CC chemokines family in monocyte and primary human macrophages [16, 17]. Application of CTRP1 led to more macrophages infiltration in atherosclerotic plaques [17]. The above studies implied that CTRP1 has a novel autocrine/paracrine regulation of monocytes/macrophages function. Using flow cytometry or computational methods, microglia/macrophages are demonstrated to be the main infiltrating immune cells in GBM and their accumulation was associated with the tumor grade and inversely correlated with patient survival [29, 34, 35]. Glioma cells can generate CCL2 to recruit monocytes/macrophages to the tumor site [1]. We subsequently analyzed and found the positive correlation of CTRP1 with tumor-infiltrating macrophages and CCL2 in GBM using TIMER (Figure 3). In addition, there is a negative correlation between CTRP1 expression and tumor purity (Figure 3(a)), which suggested CTRP1 can be produced in the tumor microenvironment [28]. Our results implied that CTRP1 may modulate CCL2 to participate in macrophages infiltration into GBM. Previous study reported that glioma cells may recruit neighboring microglia by secreting low levels of CCL2 and enhance the amplified release of CCL2 in microglia cells, which lead to recruiting more microglial cells into the tumor site to promote the progression and development of glioma [36]. Whether CTRP1 participates in the interaction between GBM and microglia/macrophages needs to be clarified in the further research.

Accumulating evidence has identified the prognostic significance of tumoral adipokines in cancer [8, 12]. In the present study, the prognostic value of CTRP1 expression in GBM was firstly evaluated using SurvExpress. SurvExpress, the widely used online biomarker validation tool, can provide effective risk assessment and survival analysis [30, 37–39]. The chosen TCGA dataset (Glioblastoma) has large samples (N=538) for more reliable results of survival analysis. Here, the higher expression of CTRP1 was found to be significantly correlated with higher risk and shorter OS time from SurvExpress (Figure 4). Therefore, CTRP1 may act as an independent predictor for GBM. Whether CTRP1 can act as a potential therapeutic target for GBM requires examining in the future. To further confirm our prediction, we detected the CTRP1 expression in human GBM tissues, and higher expression of CTRP1 was observed in tumor tissues compared with normal tissues. We also found that CTRP1 knockdown significantly inhibited cell proliferation and migration in human GBM cells. The regulation of cell proliferation by CTRP1 has been observed in chondrocytes, but there is no report in tumors [40]. Moreover, consistent with the expected results, siCTRP1 inhibited CCL2 expression, and further functional experiments confirmed that CCL2 overexpression reversed cell proliferation and migration inhibition induced by siCTRP1. Our in vitro results confirmed that CTRP1 might promote tumor progression by regulating CCL2 expression. However, the role of CTRP1 cancer promotion in human tumors deserves further study.

In summary, by using five independent and free online tools, our study provided novel insight into the expression and potential roles of CTRP1 in GBM. Our data demonstrated that the high expression of CTRP1 in GBM may be an independent predictor for GBM, and CTRP1 may contribute to the macrophages infiltration into GBM. Moreover, we observed that knockdown of CTRP1 inhibited cell proliferation and migration, suggesting the oncogene role of CTRP1 in human GMB.

## Data Availability

The data of this study are available. Readers can access the data from the manuscript or access them by contacting the corresponding author.

## Supplementary Materials

Supplementary Materials Figure S1: the correlation of the mRNA expression levels between CTRP1 and CCL2 using the “correlation” module of TIMER after tumor purity option was adjusted. Figure S2: the correlation of the mRNA expression levels between CTRP1 and CCL2 using the “correlation” module of TIMER after age option was adjusted. Figure S3: the box plot image showed mRNA levels of CTRP8 in GBM tissues (T) (N=163) and nontumor tissues (N) (N=207) from GEPIA (http://gepia.cancer-pku.cn/index.html). The datasets (“TCGA tumors vs TCGA normal + GTEx normal”) were selected for the differential analysis. According to the instructions of GEPIA, one-way ANOVA was used for the differential analysis, and the disease state (Tumor or Normal) was used as a variable for calculating differential expression. A* P* < 0.05 was considered statistically significant.
