# Supplementary material for: Identification of CTRP1 as a Prognostic Biomarker and Oncogene in Human Glioblastoma
Source: Biomed Res Int. 2019 Apr 30;2019:2582416. doi: 10.1155/2019/2582416 (PMC6515110; doi:10.1155/2019/2582416)
Supplement: Supplementary Materials — Figure S1: the correlation of the mRNA expression levels between CTRP1 and CCL2 using the “correlation” module of TIMER after tumor purity option was adjusted. Figure S2: the correlation of the mRNA expression levels between CTRP1 and CCL2 using the “correlation” module of TIMER after age option was adjusted. Figure S3: the box plot image showed mRNA levels of CTRP8 in GBM tissues (T) (N=163) and nontumor tissues (N) (N=207) from GEPIA (http://gepia.cancer-pku.cn/index.html). The datasets (“TCGA tumors vs TCGA normal + GTEx normal”) were selected for the differential analysis. According to the instructions of GEPIA, one-way ANOVA was used for the differential analysis, and the disease state (Tumor or Normal) was used as a variable for calculating differential expression. A P < 0.05 was considered statistically significant. [file 2582416.f1.pdf]

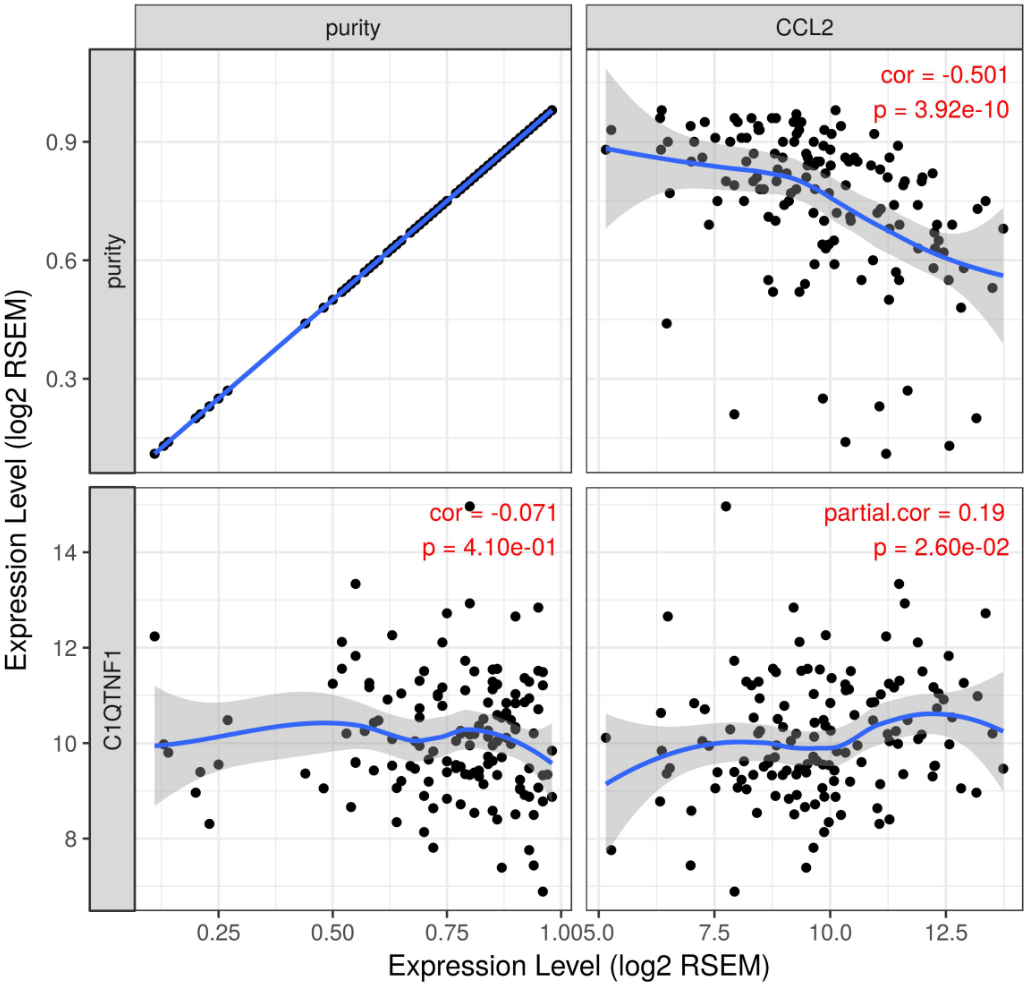

Fig. S1 The correlation of the mRNA expression levels between CTRP1 and CCL2 using the "correlation" module of TIMER after tumor purity optional adjusted.

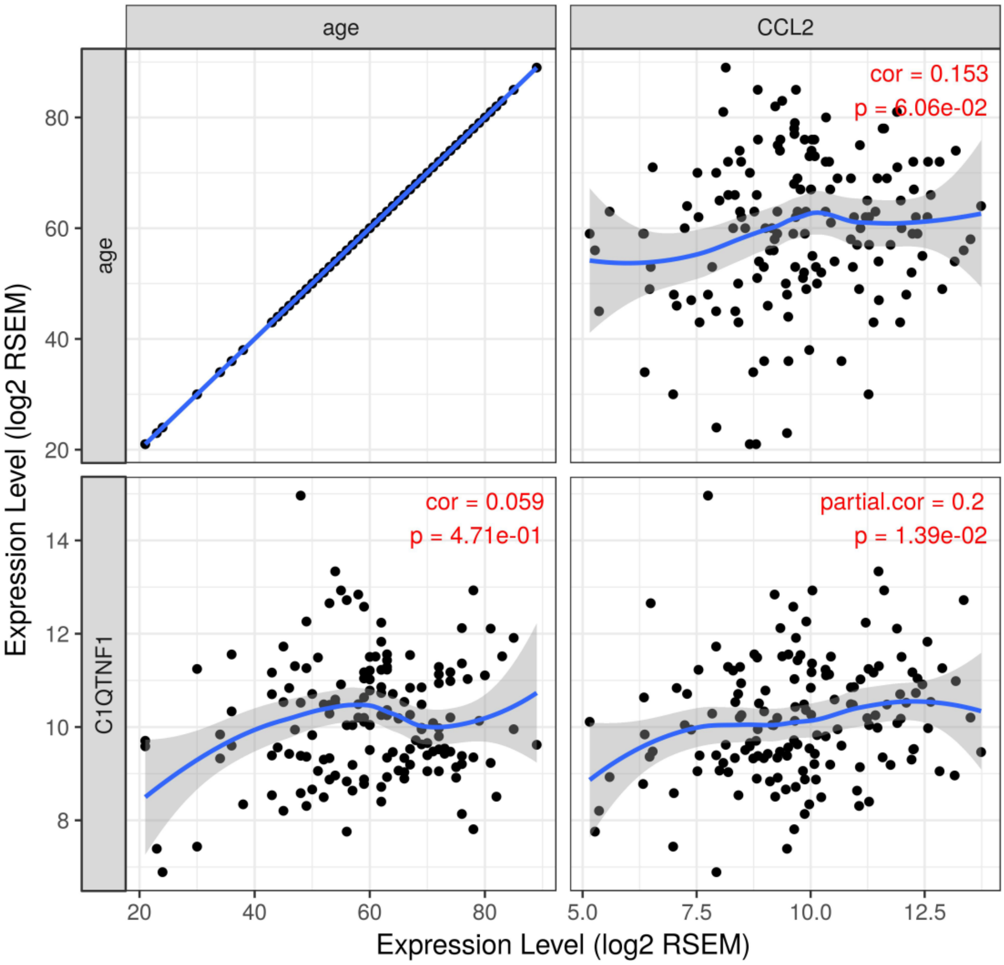

Fig. S2 The correlation of the mRNA expression levels between CTRP1 and CCL2 using the "correlation" module of TIMER after age optional adjusted.

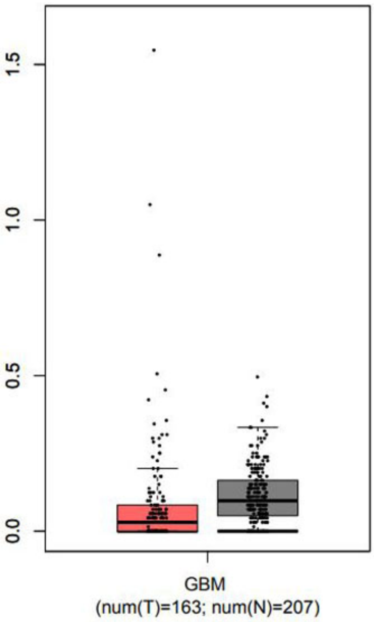

Fig. S3 The box plot image showed mRNA levels of CTRP8 in GBM tissues (T)(N=163) and non-tumor tissues (N) (N=207) from GEPIA (http://gepia.cancer-pku.cn/index.html). The datasets ("TCGA tumors vs TCGA normal + GTEx normal") were selected for the differential analysis. According to the instructions of GEPIA, one-way ANOVA was used for the differential analysis, and the disease state (Tumor or Normal) was used as variable for calculating differential expression. A $P < 0.05$ was considered statistically significant.
